# Facility-Level Approaches for COVID-19 When Caseload Surpasses Surge Capacity

**DOI:** 10.4269/ajtmh.20-0681

**Published:** 2020-06-26

**Authors:** David A. Walton, Louise C. Ivers

**Affiliations:** 1 Department of Global Health and Social Medicine, Harvard Medical School, Boston, Massachusetts;; 2 Division of Global Health Equity, Brigham and Women’s Hospital, Boston, Massachusetts;; 3 Build Health International, Boston, Massachusetts;; 4 Center for Global Health, Massachusetts General Hospital, Boston, Massachusetts

## Abstract

As COVID-19 cases continue to increase globally, fragile health systems already facing challenges with health system infrastructure, SARS-CoV-2 diagnostic capacity, and patient isolation capabilities may be left with few options to effectively care for acutely ill patients. Haiti—with only two laboratories that can perform reverse transcriptase PCR for SARS-CoV-2, a paucity of hospital beds, and an exponential increase in cases—provides an example that underpins the need for immediate infrastructure solutions for the crisis. We present two COVID-19 treatment center designs that leverage lessons learned from previous outbreaks of communicable infectious diseases and provide potential solutions when caseload exceeds existing capacity, with and without access to SARS-CoV-2 testing. These designs are intended for settings in which health facilities and testing resources for COVID-19 are surpassed during the pandemic, are adaptable to local conditions and constraints, and mitigate the likelihood of nosocomial transmission while offering an option to care for hospitalized patients.

Several low- and middle-income countries have slowed transmission of COVID-19 through rapid implementation of physical distancing policies; public health investment in testing, tracing, and isolating; and the mobilization of existing health workers.^1–3^ However, COVID-19 cases continue to increase in countries with fragile health systems, and lack of testing capacity and clinical capacities to care for ill patients may become barriers to providing effective care.^4^ Haiti, a country in which we have both worked as clinicians for over a decade, puts these needs in sharp relief.^5^ At the time of this writing, PCR-confirmed cases of COVID-19 in Haiti are rapidly escalating, and they likely represent only a fraction of actual cases—the only two laboratories in the country with the ability to perform SARS-CoV-2 reverse transcription-PCR (RT-PCR) being overwhelmed with requests.^6,7^ Other diagnostic modalities, such as the SARS-CoV-2 GeneXpert test, remain difficult to access, and rapid immunoassays have thus far proven to have limited or no utility for patient care.^8,9^ Modeling by Cornell University and Oxford University projects up to 430,000 people in Haiti might require hospitalization, which would require more than 9,000 additional hospital beds^10^; however, fewer than 300 hospital beds are currently available for COVID-19 patients.^11^ Hospitals are already beginning to report an inability to deal with the significant influx of patients. Lack of access to hospital beds, let alone critical care beds, will likely continue to be a major bottleneck to the care of patients. Haiti offers one example, but other countries have experienced similar challenges with vulnerable populations, often leveraging unique solutions to add additional bed capacity for COVID-19 patients.^12,13^

In March, the WHO released guidelines on the establishment of treatment centers for severe acute respiratory infections.^14^ The document is a thorough and welcome guide, created as a response to the COVID-19 pandemic, but the infrastructure solutions proposed are likely to be out of reach for large portions of the global population living where health budgets are woefully inadequate to ensure safe delivery of health services in the midst of this pandemic. We have argued for more than a decade for additional health system investment as an approach to delivering global health equity; however, in Haiti, the immediate gap between available hospital beds and the projected need for COVID-19 hospitalizations seems insurmountable. Any chance at caring for vast quantities of patients sick with respiratory illness will require a simple but effective model that can be implemented by local teams with limited equipment, resources, and testing capacity.

To respond to the immediate crisis facing health workers and patients, we propose a COVID-19 treatment center design (Figure 1) that harnesses lessons learned from other outbreaks and adheres to infection prevention and control principles recommended by the WHO for the novel coronavirus. Although viral hemorrhagic fevers or diarrheal diseases have different transmission dynamics from SARS-CoV-2, principles of infection control and management of large quantities of patients in low-resource settings can carry through to COVID-19. These principles include 1) screen anyone who presents at the facility with any complaint, 2) test patients for infection if they meet certain criteria, 3) isolate individuals with confirmed infection in private rooms or cohort in wards separated from noninfected patients, and 4) have all staff inside the facility in personal protective equipment. Cholera treatment centers, typically set up for large outbreaks, cohort patients who meet the clinical case definition for cholera, and maintain stringent borders between the cholera- and non-cholera areas of health facilities.^15^ A similar cohorting approach was used for Ebola treatment centers in the 2014–2016 Ebola epidemic, and although testing for Ebola virus disease was an important component of triage, it was not always rapidly available.^16^

**Figure 1.**
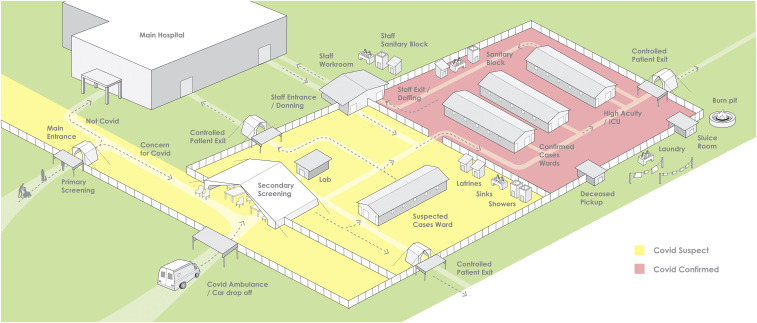
COVID treatment center campus with access to confirmatory SARS-CoV-2 testing.

Our design acknowledges a stark reality: in some low-resource settings, the volume of acute respiratory illness cases may surpass surge capacity. The design assumes that two thresholds have been reached: first, the health center no longer has space to individually isolate COVID-19 patients, and second, laboratory capacity is limited or surpassed, such that rapid, accurate testing for COVID-19 may not be available, as is the reality facing our colleagues in Haiti.

The COVID-19 treatment center is modular for rapid construction and designed to be adaptable to local conditions and constraints. The number of wards and overall footprint is flexible to adapt to the burden of disease and pragmatic budgetary constraints. Similarly, the design allows for use of local construction materials: walls can be made with lumber and plywood or with concrete blocks for a more permanent structure; large tents can also be used for wards within the campus plan. Nosocomial transmission can be mitigated with natural ventilation, which can often achieve or exceed 12 air changes per hour, and fans can be installed to improve thermal comfort for patients.^17,18^ Ideally, but not necessarily, treatment centers would be located adjacent to existing medical facilities to leverage existing water and power supplies, and allow for the ability to care for non-COVID-19 patients.

In this design, concern for nosocomial transmission would remain high in the “suspect ward,” where patients who may ultimately test positive for COVID-19 mix with patients who may have malaria, tuberculosis, or another disease that presents similarly.^19–21^ However, the design assumes that the ability to isolate suspect cases in individual rooms has been superseded by the number of cases. The risk of nosocomial infection in such wards can be mitigated (though not eliminated) by spacing beds at least 1.5 m apart and placing protective barriers between beds to reduce the spread of droplet nuclei.^22^ If caseload supersedes bed capacity, a common pitfall is to place additional beds in the ward, but this should be avoided and priority given to preserving 1.5 m between beds, which allows the maximum number of beds within the dimensions of the space allocated while aiming to mitigate nosocomial spread. If feasible, patients not requiring oxygen can also wear surgical masks.

The risk of nosocomial spread highlights the imperative to increase access to rapid, sensitive, and specific low-cost tests for SARS-CoV-2. In Haiti, testing capacity for RT-PCR has already exceeded capacity. Unable to rapidly scale up testing, many facilities have started using a clinical case definition for the diagnosis of COVID-19 even for hospitalized patients. While triaging testing resources has been used in other settings in which demand for testing exceeds capacity, including New York, California, and Washington, having unknown COVID-19 status for hospitalized patients presents special challenges.^23,24^ Figure 2 demonstrates how the design and flow would change in a situation in which no COVID-19 testing is available and cohorting of patients is instead achieved by case definition. With this design, the risk of nosocomial infection remains high but may be reduced with mitigation techniques used in the “suspect ward” (Figure 3).

**Figure 2.**
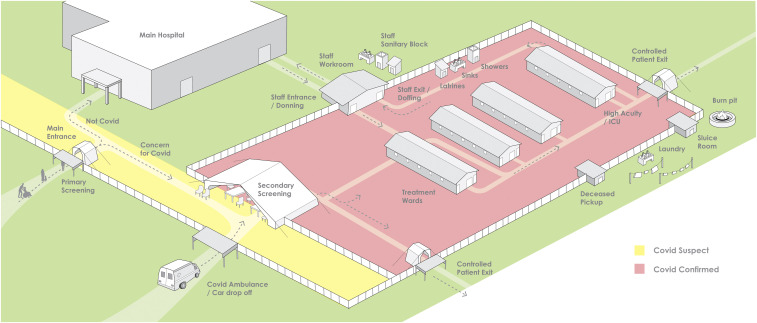
COVID treatment center campus without access to confirmatory SARS-CoV-2 testing.

**Figure 3.**
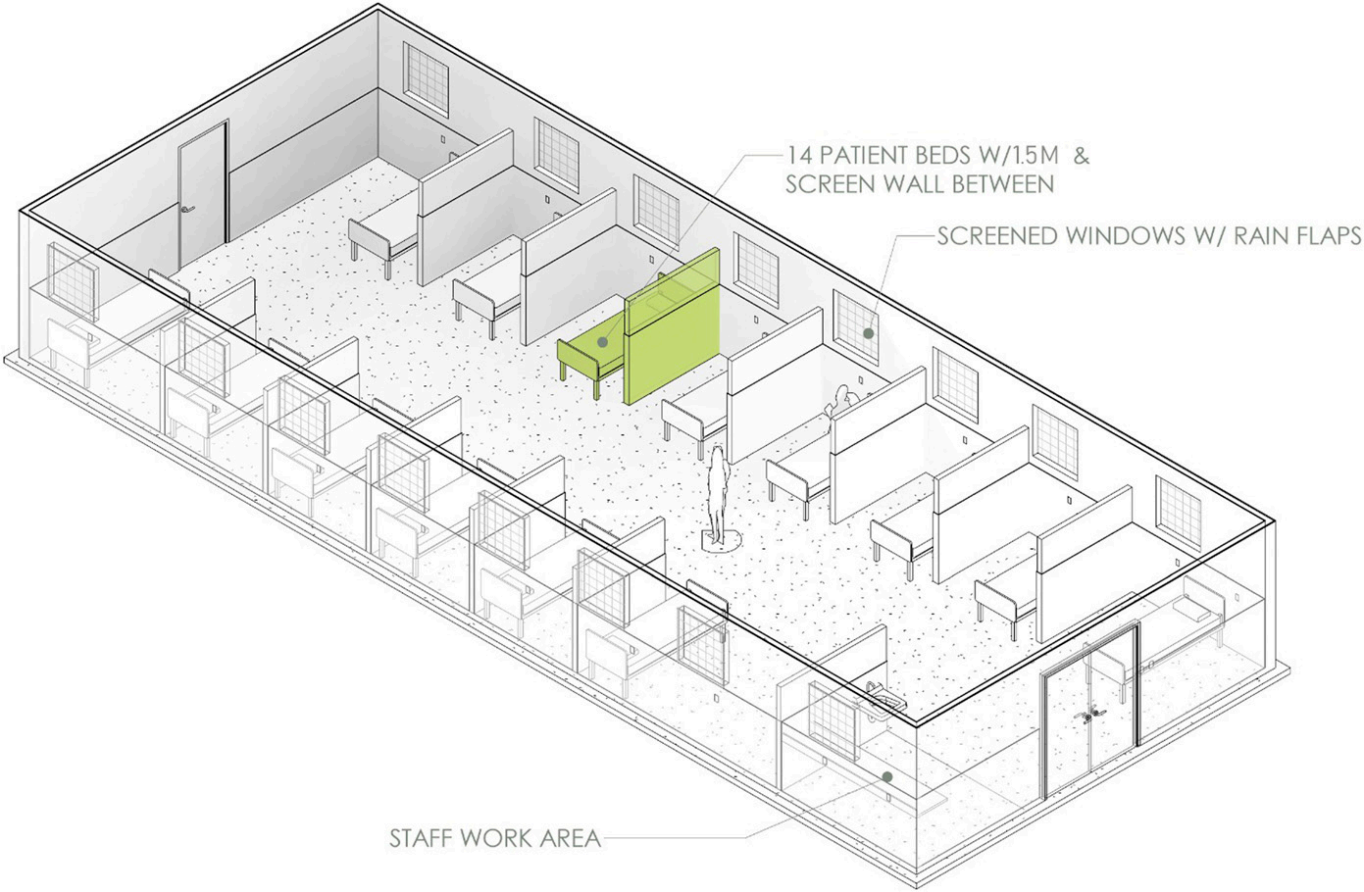
Suspect ward design.

As we have seen in Haiti, the rapid surge of COVID-19 in countries with fragile health systems has far surpassed the existing health infrastructure’s ability to offer safe spaces to care for patients, with many countries yet to hit their peak caseload. Without the ability to effectively isolate COVID-19 patients or suspect patients who need hospitalization, we would expect a disproportionately higher morbidity and mortality than in other settings with capacity to receive a surge of cases. As one component of the emergency response, COVID-19 treatment centers harness lessons learned from previous infectious disease outbreaks to cohort COVID-19 patients who require hospitalization, mitigate (but not eliminate) the risks of nosocomial transmission, and streamline care.
